# Supplementary material for: High-performance near-infrared OLEDs maximized at 925 nm and 1022 nm through interfacial energy transfer
Source: Nat Commun. 2024 May 31;15:4664. doi: 10.1038/s41467-024-49127-x (PMC11143248; doi:10.1038/s41467-024-49127-x)
Supplement: Supplementary file 1 — Supplementary Information [file 41467_2024_49127_MOESM1_ESM.pdf]

Supplementary Information

High-Performance Near-infrared OLEDs Maximized at 925 nm and 1022 nm through Interfacial Energy Transfer

Chieh-Ming Hung[1,6], Sheng-Fu Wang[1, 6], Wei-Chih Chao[1, 6], Jian-Liang Li[1], Bo-Han Chen[2], Chih-Hsuan Lu[2], Kai-Yen Tu[1], Shang-Da Yang[2], Wen-Yi Hung[3], Yun Chi[4]*, Pi-Tai Chou[1,5]*

[1]Department of Chemistry, National Taiwan University, Taipei, Taiwan, R.O.C.

[2]Institute of Photonics Technologies, National Tsing Hua University, Hsinchu, Taiwan

[3]Institute of Optoelectronic Sciences, National Taiwan Ocean University, Keelung, Taiwan

[4]Department of Materials Sciences and Engineering and Department of Chemistry, City University of Hong Kong, Hong Kong SAR, China

[5]Center for Emerging Materials and Advanced Devices, National Taiwan University, Taipei, Taiwan

[6]These authors contributed equally.

*E-mail: yunchi@cityu.edu.hk; chop@ntu.edu.tw

Supplementary Note 1 | Experimental section

Chemicals: PDMS stamps were prepared from the oligomer Silgard 184A and the curing agent Silgard B. PEDOT:PSS, Clevios™ P VP AI 4083, was purchased from Heraeus. Dipyrazino[2,3-f :2',3'-h]quinoxa line-2,3,6,7,10,11-hexacarbonitrile (HATCN, 99%), N,N'-Bis(naphthalen-1-yl)-N,N'-bis(phenyl)-benzidine (NPB, 99%), 1,3-Bis(N-carbazolyl)benzene (mCP, 99%), 1,3,5-Tris(1-phenyl-1Hbenzimidazol-2-yl)benzene (TPBi, 99%), Lithium fluoride (LiF, 99.99%), Poly(9-vinylcarbazole) (PVK, M_W >20,000), 2,2'-((2Z,2'Z)-((12,13-bis(2-butyloctyl)-3,9-dinonyl-12,13-dihydro-[1,2,5]thiadiazolo[3,4-e]thieno[2",3":4',5']thieno[2',3':4,5]pyrrolo[3,2-g]thieno[2',3':4,5]thieno[3,2-b]indole-2,10-diyl)bis(methanylylidene))bis(5,6-dichloro-3-oxo-2,3-dihydro-1H-indene-2,1-diylidene))dimalononitrile (BTP-eC9, 99%), 2,2'-((2Z,2'Z)-((12,13-Bis(2-ethylhexyl)-3,9-diundecyl-12,13-dihydro-2-(2-ethylhexyl)- [1,2,3]triazole[3,4-e]thieno[2” ,3":4',5']thieno-[2',3':4,5]pyrrolo[3,2-g] thieno[2',3':4,5]thieno-[3,2-b]indole-2,10-diyl)bis(methanylylidene))bis-(5,6-difloro-3-oxo-2,3-dihydro-1H-indene-2,1-diylidene))dimalononitrile (Y11, 99%), C_{60} (99%), Bathocuproine (BCP, 99%), and 2,4,6-Tris[3-(diphenylphosphinyl)phenyl]-1,3,5-triazine (PO-T2T, 99%) was purchased from Lumtec. Chlorobenzene (CB, anhydrous, 99.5%) and Chloroform (CF, anhydrous, 99.5%) were purchased from Sigma Aldrich.

Supplementary Note 2 | Setup of light source and transient absorption spectroscopy

The measurements were performed using a commercial Yb:KGW laser system (Pharos, Light Conversion) with a central wavelength of 1030 nm, an average power of 2.5 W, a repetition rate of 3.125 kHz, a pulse energy of 800 μJ and a pulse duration of 190 fs. Two identical pulses were produced with a low-GDD 50/50 beam splitter and passed through our designed nonlinear compressor using a previously reported technique, namely, multiple plate compression (MPC). For this experiment, a high-pass filter with a cut-off wavelength of 980 nm was applied. Pulse compression was achieved with 8 chirped mirror bounces (Ultrafast Innovation), thus removing the material dispersion introduced by the optics before the sample. The pump pulse was modulated by a laser-triggered mechanical chopper modulating at half the laser repetition rate (1.5625 kHz). A broadband half-wave plate and wire-grid polarizer were used to precisely control the excitation power and ensure that no nonlinear effects were introduced. The delay time (relative to the pump pulse) of the probe pulse was adjusted by a linear translation stage (DL325, Newport) that supports a delay range up to ~2.2 ns. The pump and probe beams were both focused on the sample in a noncollinear manner with a cross-angle of 5 degrees. Different focusing conditions were chosen for pump and probe pulses to ensure that the focused pump spot size (~67.7 μm) was slightly larger than the focused probe spot size (~27.3 μm) and that the probed region was uniformly excited. After the pulses passed through the sample, the transmitted probe pulse was spatially separated and guided into our designed spectrometer, which includes a high-speed linear array camera (Glaz Linescan-I-Gen2, Synertronic with S12198-512Q CMOS, Hamamatsu) to ensure that each probe pulse is captured. Since the pump pulses are modulated at half of the repetition rate, the spectral difference between every two probe shots (one sees the pump, while the other does not) provides the $\Delta T/T$ signal.

Supplementary Note 3 | Synthesis of BTPV-eC9

A mixture of BTPV-eC9-CHO[1] (200 mg, 0.18 mmol), 2-(5,6-dichloro-3-oxo-2,3-dihydro-1H-inden-1-ylidene)malononitrile (2ClIC) (232 mg, 0.88 mmol) in chloroform (20 mL) was added pyridine (1.0 mL) under nitrogen. The reaction mixture was stirred at room temperature 16 hours. The mixture was then purified by column chromatography using CH_2Cl_2 to give BTPV-eC9 as a dark blue solid. (125 mg, 44% yield). ^{1}H NMR (500 MHz, $CDCl_3$): δ 8.76 (s, 2H), 8.69-8.60 (m, 2H), 8.53 (d, J = 11.7 Hz, 2H), 7.93 (s, 2H), 7.75 (d, J = 14.2 Hz, 2H), 4.71-4.59 (m, 4H), 3.03 (t, J = 7.8 Hz, 4H), 2.15- 2.06 (m, 2H), 1.90-1.83 (m, 4H), 1.50-1.45 (m, 4H), 1.41-1.36 (m, 4H), 1.32-1.25 (m, 20H), 1.15-0.86 (m, 34H), 0.73-0.65 (m, 12H). ^{13}C NMR (126 MHz, $CDCl_3$): δ 187.36, 157.40, 147.49, 145.36, 144.71, 143.29, 139.63, 139.36, 138.72, 137.76, 137.17, 136.48, 133.14, 128.29, 128.22, 127.01, 125.02, 123.45, 122.52, 114.52, 114.47, 113.23, 69.48, 55.46, 38.90, 31.91, 31.59, 30.41, 30.34, 30.26, 30.20 ,29.80, 29.76, 29.55, 29.51, 29.42, 29.40, 29.35, 28.74, 28.04, 27.88, 25.41, 25.24, 22.82, 22.78, 22.72, 22.55, 22.53, 14.17, 14.06, 13.83, 13.80. HR-MALDI *m/z* calcd. for $[M]^{\bullet+}$ $C_{90}H_{98}Cl_4N_8O_2S_5$ 1622.5170, found 1625.5147. For the synthesis route, please refer to Supplementary Fig. 24

Supplementary Note 4 | Cyclic voltammetry (CV) measurements

Cyclic voltammetry (CV) measurements were conducted using an Autolab PGSTAT30 with a scanning rate of 50mV/s, at room temperature. The three-electrode cell consisted of a platinum disk, an Ag/AgCl wire and a platinum wire counter were used as working, reference and counter electrode, respectively. Ferrocene and BTPV-ec9 were dissolved in dichloromethane solution of tetrabutylammonium hexafluorophosphate (n-Bu_4NPF_6, 0.1M) at the concentration of 10^{-3}M.

Supplementary Note 5 | Device fabrication of BTPV-eC9 OLEDs:

The BTP-eC9 OLED were fabricated with the device structure of ITO/PEDOT:PSS (15 nm)/ BTPV-eC9 (~ 100 nm)/PFN-Br(~ 5 nm)/Ag (120 nm). After the UV−ozone treatment for 20 min, PEDOT:PSS spin-coated at 5000 rpm for 30 s on ITO and annealed 150 °C for 20 min. Then transferred into an N_2-filled glove box (< 0.1 ppm O_2 and H_2O). After film cooled down to room temperature, BTPV-eC9 (18 mg mL^{-1} in CF) and was spin-coating condition of 3000 rpm with the hot solution. PFN-Br (0.5 mg mL^{-1} in MeOH) and was spin-coating drop of 5000 rpm. 120 nm silver electrode were evaporated under high vacuum (< 1×10^{-6} Torr) sequentially.

Supplementary Note 6 | Device fabrication of Pt(II) No.2/BTPV-eC9 stamp OLEDs

The fabrication process involves the sequential deposition on ITO glass of HATCN (10 nm)/NPB (50 nm)/mCP (15 nm)/Pt(II) No.2 (4nm). Subsequently, BTPV-eC9 is spin-coated onto PDMS, which has undergone UV-ozone treatment. It is crucial to note that the transfer quality of PDMS directly influences the performance of the OLED. Finally, PO-T2T (25 nm)/LiF (1.5 nm)/Al (120 nm) is deposited through thermal evaporation.

Supplementary Note 7 | Emission lifetime and FRET analysis

The emission decays were analyzed by the sum of exponential functions and convoluted with the instrument response function (IRF), which allowed partial elimination of instrument time broadening and thus rendered a temporal resolution of ~ 300 ps.

$$I(t) = \sum_{i=1}^{n} a_i e^{-\tau/\tau_i}$$

where a_i and τ_i are the amplitudes and decay times of the n exponential components of the fluorescence decay.

To obtain useful structure information from energy transfer, the measured efficiency must be related to the distance between the two fluorophores. FRET efficiency, E can be obtained by measuring the fluorescence intensity.

$$E = \frac{R_0^6}{R_0^6 + r^6} = 1 - \frac{F_{DA}}{F_D}$$

Where F_{DA} is the fluorescence of donor with acceptor, F_D is the fluorescence of donor without acceptor, and the Forster radius R_0 is the distance between donor and acceptor fluorophore for 50% FRET efficiency.

The Förster radius (in Ångstrom) can be found using:

$$R_0 = \left(\frac{9000(ln\,10)\kappa_p^2 Q_D}{N_A 128\pi^5 n_D^4} I\right)^{1/6} = 8.79 \times 10^{-5} \times \kappa^2 \times Q_D \times n^{-4} \times I(\lambda)$$

Where Q_D is the quantum yield of the donor, N_A is Avogadro's number, n_D is refractive index of the medium, κ^2 is defined as

$$\kappa^2 = (cos\,\theta_T - 3\,cos\,\theta_D\,cos\,\theta_A)^2$$

where θ_T is the angle between the donor emission transition dipole moment and the acceptor absorption transition dipole moment, and θ_D and θ_A are the angles between the donor-acceptor connection line and the donor emission and the acceptor absorption transition dipole moments, respectively. In general, κ^2 = 2/3 for randomly oriented dipoles and varies between 0 and 4 for the cases of orthogonal and parallel dipoles.

The overlap integral (I) defined as

$$I = \int_0^\infty PL_{D-corr}(\lambda)\varepsilon_A(\lambda)\lambda^4 d\lambda$$

I is a quantitative measure of the donor-acceptor spectral overlap over all wavelengths; it is a function of the normalized donor emission spectrum ($PL_{D\text{-}corr}$), and the acceptor absorption spectrum (ε_A).

Since the emissive layer of the solid film material used in this study exhibits optical anisotropy, where the refractive index and extinction coefficient of horizontally and vertically polarized light are different, the light parameters of the material in solution should not be used directly. Moreover, the light parameters should be considered that are perpendicular to the film.

In our study, the Förster radius R_0 and parameters used in the calculations are as follows:

n = 1.3

$k^2 = 0.66$

Q_D of $Pt(fprpz)_2 = 0.786$

Q_D of Pt(II) No.2 = 0.384

For $Pt(fprpz)_2$ / BTP-eC9, $I = 1.65 x 10^{16}$, $R_0 = 7.8$ nm

For $Pt(fprpz)_2$ / Y11, $I = 6.58 x 10^{16}$, $R_0 = 9.8$ nm

For Pt(II) No.2 / BTPV-eC9, $I = 1.97 x 10^{17}$, $R_0 = 10.1$ nm

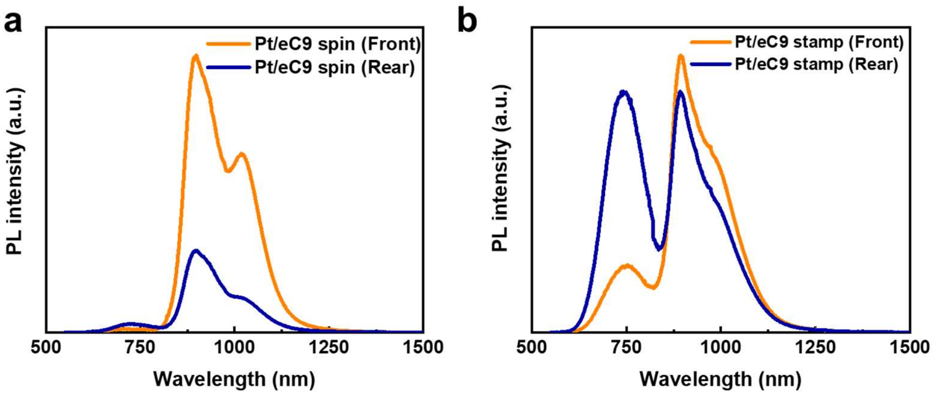

Supplementary Fig. 1 | Photoluminescence of front or rear excitation and emission. Steady-state photoluminescence spectra of (a) Pt/eC9 spin and (b) Pt/eC9 stamp, excited at 505nm.

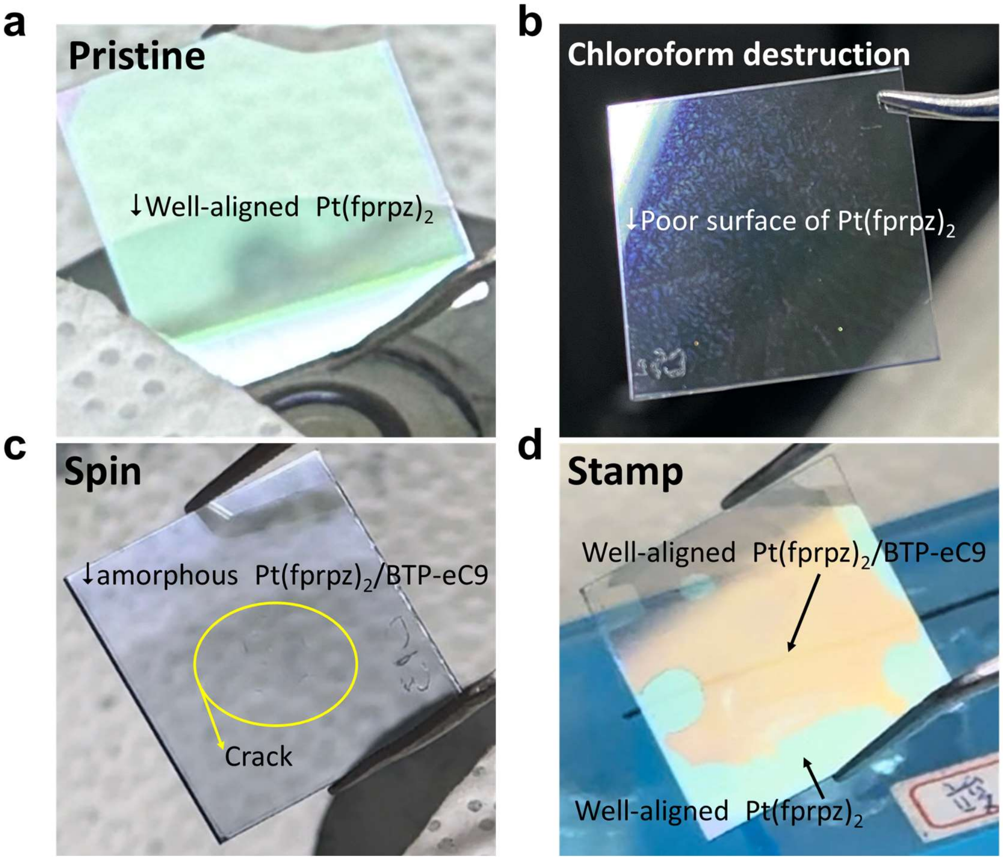

Supplementary Fig. 2 | Films after different post-processing processes, from left to right: original, spin-processed and stamped with BTP-eC9. (a) Well-aligned Pt(fprpz)$_2$ layer. (b) After the chloroform solvent rinse and spin coating process, it is destroyed into an amorphous Pt(fprpz)$_2$ film. (c) Amorphous Pt(fprpz)$_2$/BTP-eC9 bilayer affected by spin-coated BTP-eC9. (d) Well-aligned Pt(fprpz)$_2$/BTP-eC9 bilayer through imprinting process.

Supplementary Table 1 | The emission wavelength, photoluminescence lifetime, and photoluminescence quantum yield (PLQY) of different types of thin films.

Sample	Excited (nm)	λ_{em}^{max} (nm)	QY (%) of (average ± error)	Sources for obtaining PLQY [a]
BTP-eC9	800	940	5.57 (5.42 ± 0.17)	BTP-eC9
$Pt(fprpz)_2$	505	750	78.6 (77.6 ± 0.92)	$Pt(fprpz)_2$
$Pt(fprpz)_2$/ spin solvent destruction	505	735	0.92 (0.64± 0.24)	$Pt(fprpz)_2$
$Pt(fprpz)_2$/BTP-eC9 spin	800	928	5.04 (5.00 ± 0.05)	$Pt(fprpz)_2$
$Pt(fprpz)_2$/BTP-eC9 stamp	800	925	8.85 (8.61 ± 0.21)	BTP-eC9
$Pt(fprpz)_2$/BTP-eC9 spin	505	735	0.62 (0.51 ± 0.09)	$Pt(fprpz)_2$
		928	4.65 (4.53 ± 0.12)	BTP-eC9
$Pt(fprpz)_2$/BTP-eC9 stamp	505	750	7.95 (7.79 ± 0.14)	$Pt(fprpz)_2$
		925	7.12 (6.96 ± 0.15)	BTP-eC9

[a] 650-850 nm belongs to the emission of $Pt(fprpz)_2$, and 850-1200 nm belongs to the emission of BTP-eC9.

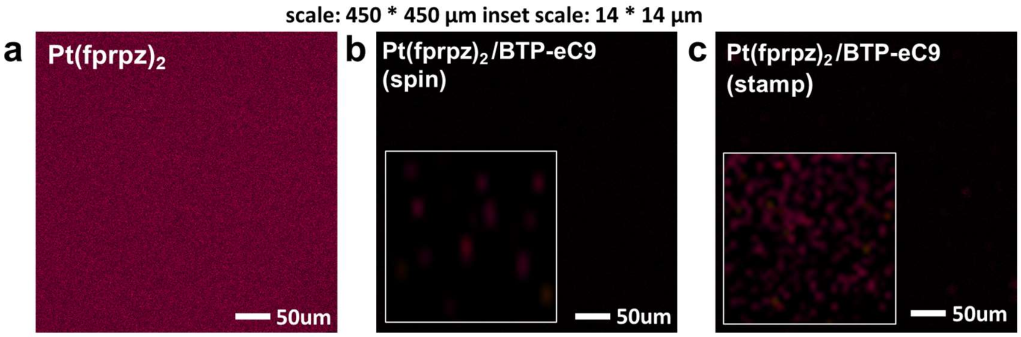

Supplementary Fig. 3 | Photoluminescence mapping of spin or stamp treated films. Confocal photoluminescence intensity maps of (a) $Pt(fprpz)_2$, (b) $Pt(fprpz)_2$/BTP-eC9 (spin) and (c) $Pt(fprpz)_2$/BTP-eC9 (stamp).

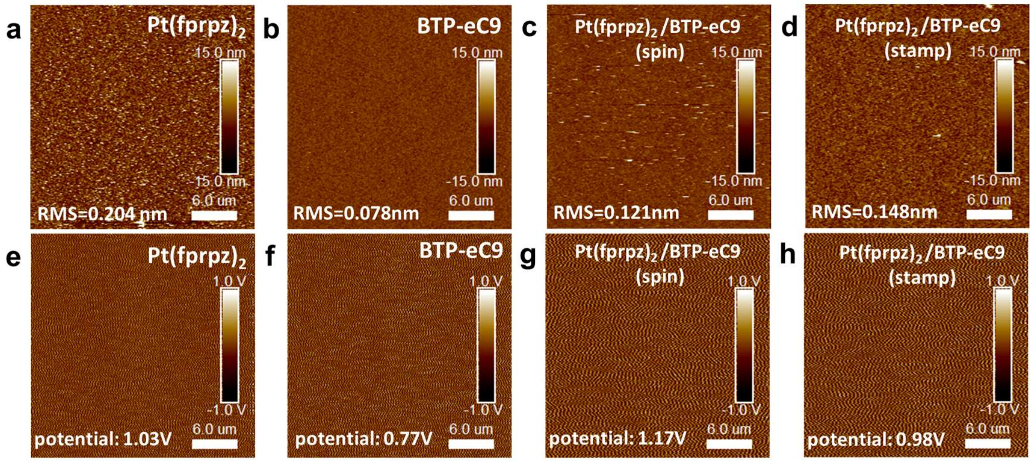

Supplementary Fig. 4 | Surface morphology and surface potential. (a-d) AFM topography and (e-h) KPFM images of Pt(fprpz)$_2$, BTP-eC9, Pt(fprpz)$_2$/BTP-eC9 (spin), and Pt(fprpz)$_2$/BTP-eC9 (stamp).

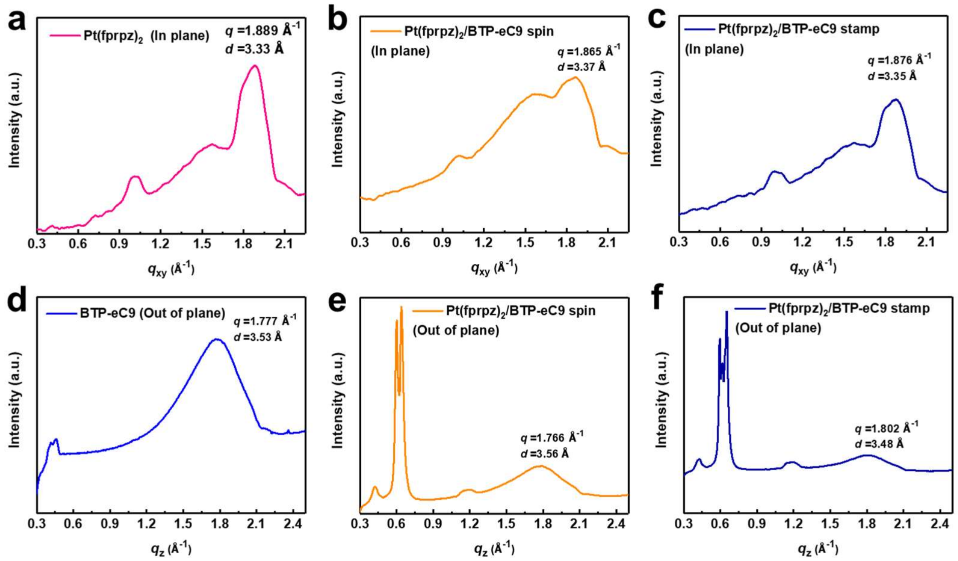

Supplementary Fig. 5 | 1D In-plane and out-of-plane x-ray diffraction fitting. (a-c) The in-plane and (d-f) out of plane profiles of GIWAXS patterns for the Pt(fprpz)$_2$, BTP-eC9, Pt(fprpz)$_2$/BTP-eC9 (spin), and Pt(fprpz)$_2$/BTP-eC9 (stamp).

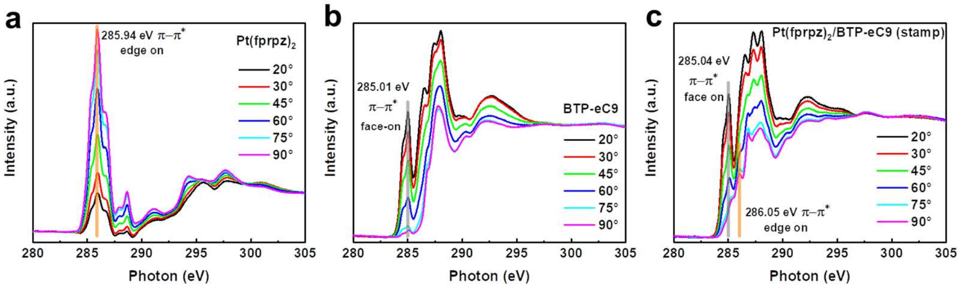

Supplementary Fig. 6 | Near-edge x-ray absorption fine structure (NEXAFS) fitting. NEXAFS spectra for (a) $Pt(fprpz)_2$, (b) BTP-eC9, and (c) $Pt(fprpz)_2$/BTP-eC9 (stamp).

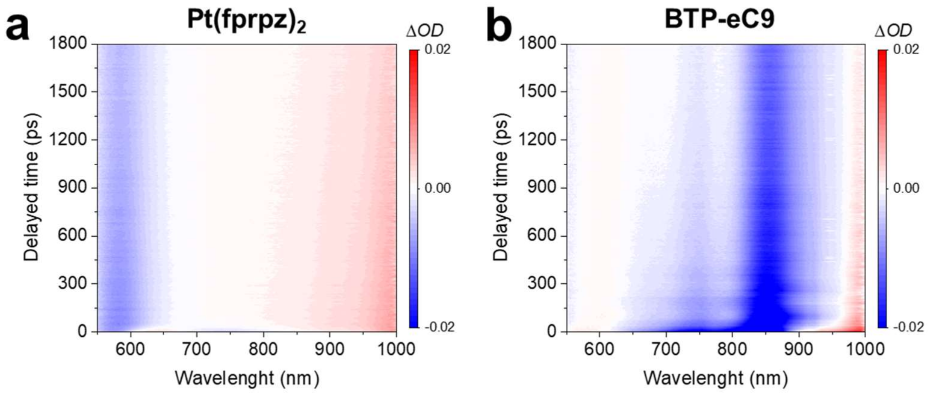

Supplementary Fig. 7 | 2D TA contour plot of neat acceptor films. Pseudo-color plots of the picosecond transient absorption (ps-TA) spectra for (a) $Pt(fprpz)_2$ film and (b) BTP-eC9 film.

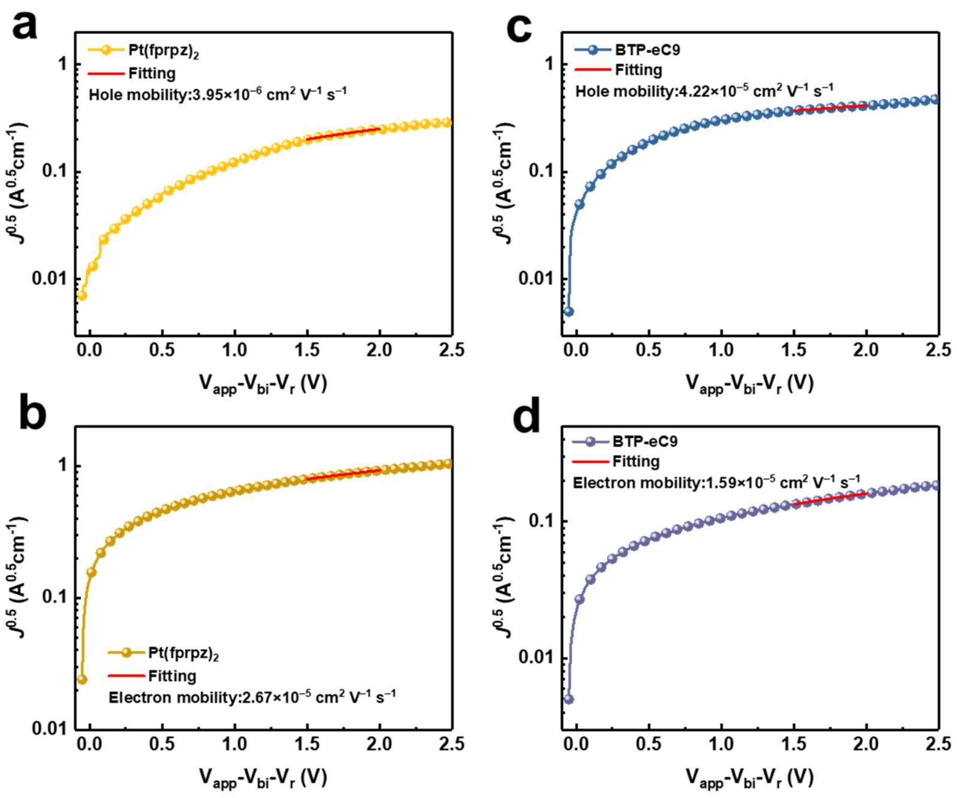

Supplementary Fig. 8 | Hole and electron mobility measurements. SCLC fitting for the $J^{0.5}$–V curve of (a) Pt(fprpz)$_2$ (b) BTP-eC9 hole-only devices and (c) Pt(fprpz)$_2$ (d) BTP-eC9 electron-only devices.

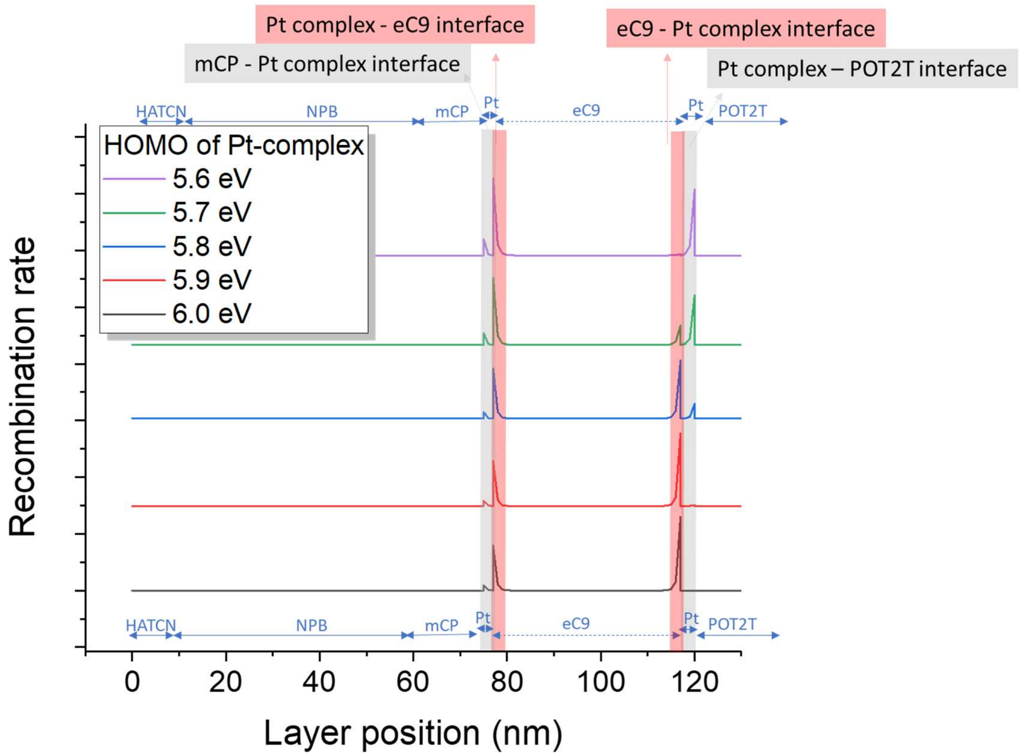

Supplementary Fig. 9 | The simulation results using Setfos. Recombination distribution in devices containing different HOMO energy levels of the Pt-complexes.

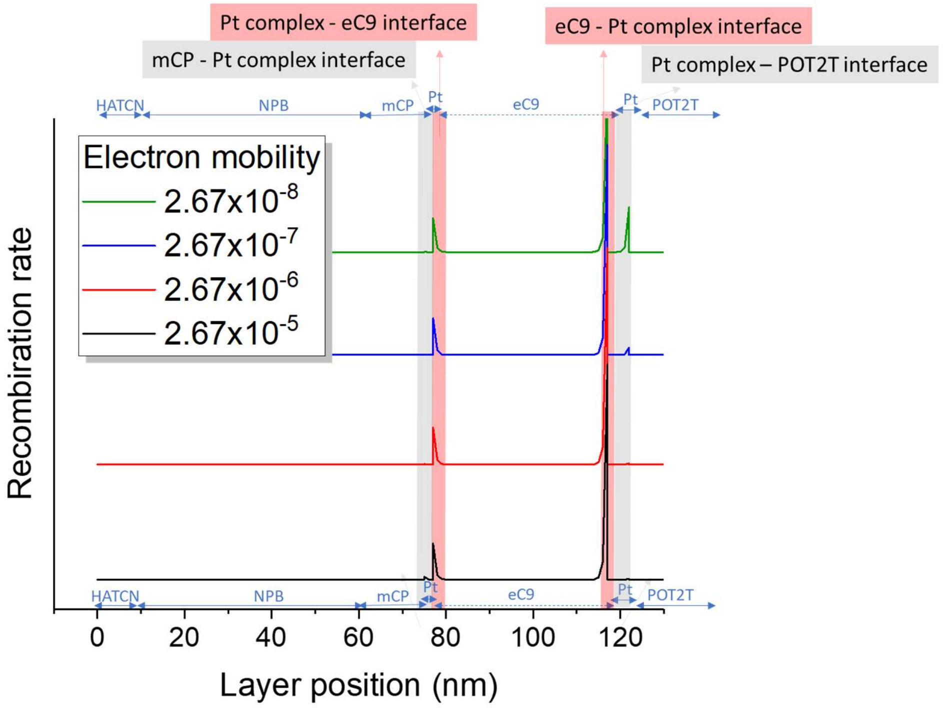

Supplementary Fig. 10 | The simulation results using Setfos. Recombination distribution in devices containing different electron mobility of the Pt-complexes.

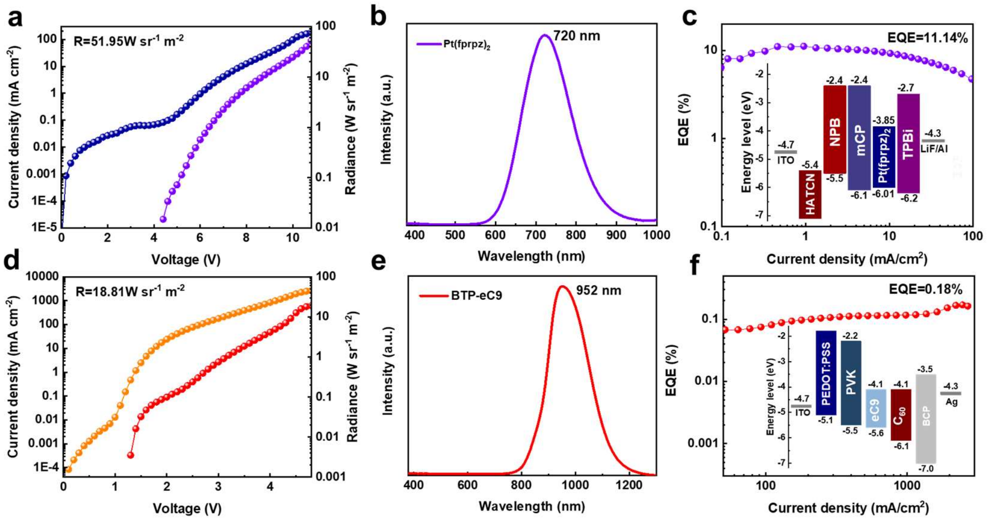

Supplementary Fig. 11 | OLEDs performance measurements. Radiance and current versus voltage curves: (a) Pt(fprpz)$_2$ and (d) BTP-eC9. EL spectra of (b)Pt(fprpz)$_2$ and (e)BTP-eC9. EQE versus current density curves with device energy level diagram: (c) Pt(fprpz)$_2$ and (f) BTP-eC9.

Supplementary Note 8 | Simulation method

Thickness-dependent recombination ratios were performed using the commercial software Setfos with an electrical simulation module. Electron and hole mobilities according to the Poole-Frenkel model were used in the simulations. The setup parameters for the electrical simulations are shown in Table S1, with excitons generated in the emissive layer and following standard Langevin recombination. The simulated specific structure consists of ITO (150 nm) / HAT-CN (10 nm) / NPB (50 nm) / mCP (15 nm) / $Pt(fprpz)_2$/BTP-eC9/ $Pt(fprpz)_2$ / PO-T2T (30 nm) / LiF (1 nm) / Al (120 nm) on a glass substrate. Simulated scan of various Pt-complex/eC9/Pt-complex multilayer thickness.

Supplementary Table 2 | Setting parameters for the electrical simulations of recombination rate.

Material	HOMO (eV)	LUMO (eV)	Electron mobility ($cm^2 V^{-1} s^{-1}$)	Hole mobility ($cm^2 V^{-1} s^{-1}$)
NPB	5.5	2.4	1.51×10^{-7}	2.60×10^{-4}
mCP	6.1	2.4	5.10×10^{-5}	1.20×10^{-4}
$Pt(fprpz)_2$[a]	6.01	3.85	2.67×10^{-5}	3.95×10^{-6}
BTP-eC9[b]	5.6	4.1	1.59×10^{-5}	4.22×10^{-5}
PO-T2T	7.5	3.5	1.00×10^{-3}	1.00×10^{-5}

[ab] See FigureS7 for SCLC analysis.

Supplementary Note 9 | Simulation results 1

Multiple zones were first identified through electrical modeling. As shown in Figure S9 the recombination of incident holes and electrons mainly occurs within the BTP-eC9 and Pt-complex-associated emissive layer but is dispersed near several interfaces of the BTP-eC9 and Pt-complex composite device. Since the increase in the effective $Pt(fprpz)_2$/BTP-eC9 recombination area contributes to the improvement of device efficiency, we first simulated Pt complexes with different thicknesses to analyze the ratio of recombination rate (relative to the total recombination area) at the BTP-eC9/$Pt(fprpz)_2$ interface. It was found that the area ratio of the effective recombination region could increase from 18% to 58% as the thickness of the $Pt(fprpz)_2$ decreases from 10 nm to 5 nm.

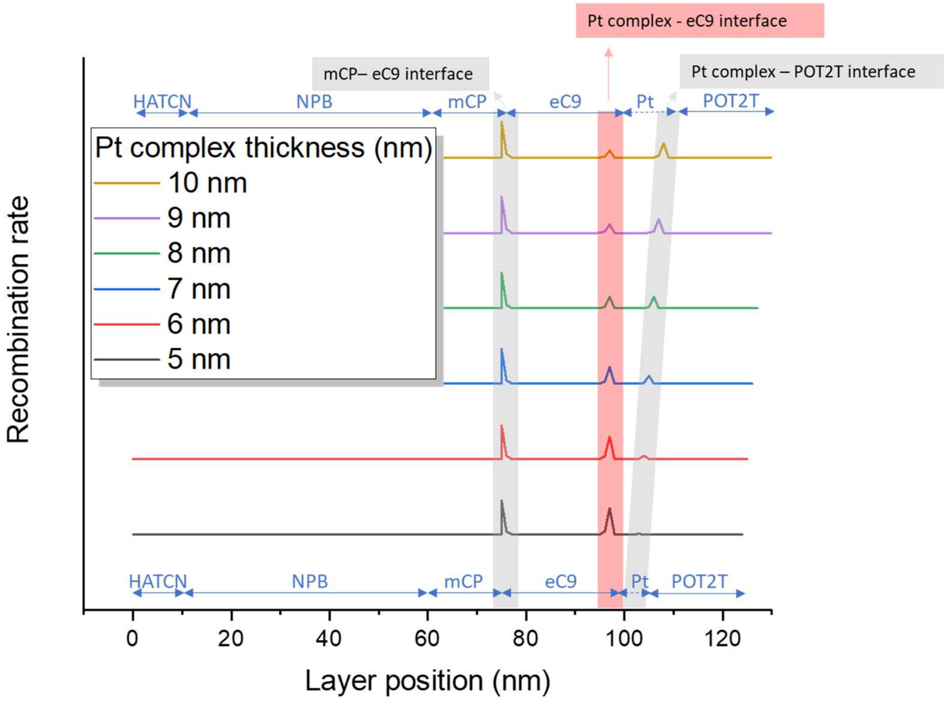

Supplementary Fig. 12 | The simulation results using Setfos. Recombination distribution in devices containing various Pt(fprpz)$_2$ thicknesses.

Supplementary Note 9 | Simulation results 2

Then the thickness of BTP-eC9 layer was adjusted and Pt(fprpz)$_2$ was added on both sides of BTP-eC9 to increase the ratio of effective Pt(fprpz)$_2$/BTP-eC9 recombination area. As shown in Figure S10, it can be clearly seen that when the thickness of BTP-eC9 increases to 40 nm, almost all recombination occurs at the interface between the two Pt(fprpz)$_2$ and BTP-eC9, and the value can reach 93% effective recombination.

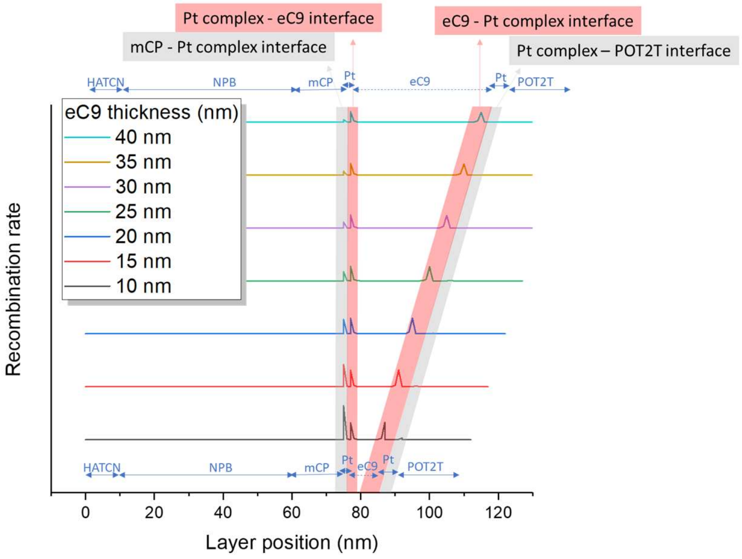

Supplementary Fig. 13 | The simulation results using Setfos. Recombination distribution in devices containing various $Pt(fprpz)_2$ thicknesses.

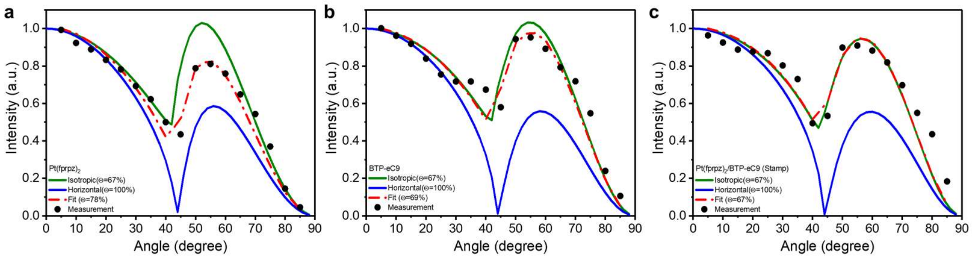

Supplementary Fig. 14 | Angle-Dependent Photoluminescence. Horizontal dipole ratios for (a) $Pt(fprpz)_2$ (b) BTP-eC9 (c) $Pt(fprpz)_2$/BTP-eC9 (stamp)

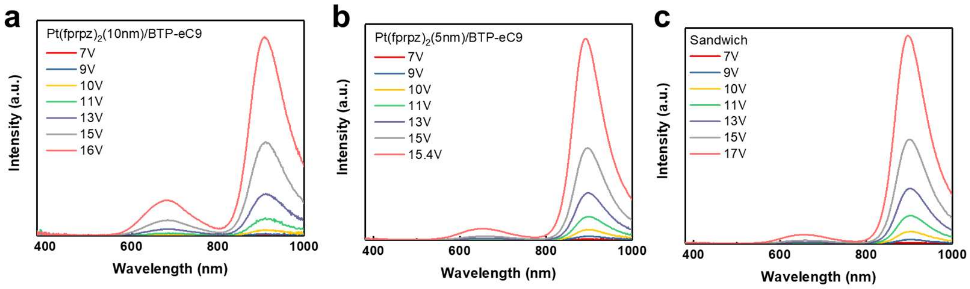

Supplementary Fig. 15 | Electroluminescence spectrum of different OLEDs structures. NIR-OLED EL spectra at different voltages (a) $Pt(fprpz)_2$ (10nm)/BTP-eC9 (b) $Pt(fprpz)_2$ (5nm)/BTP-eC9 (c) Sandwich structure.

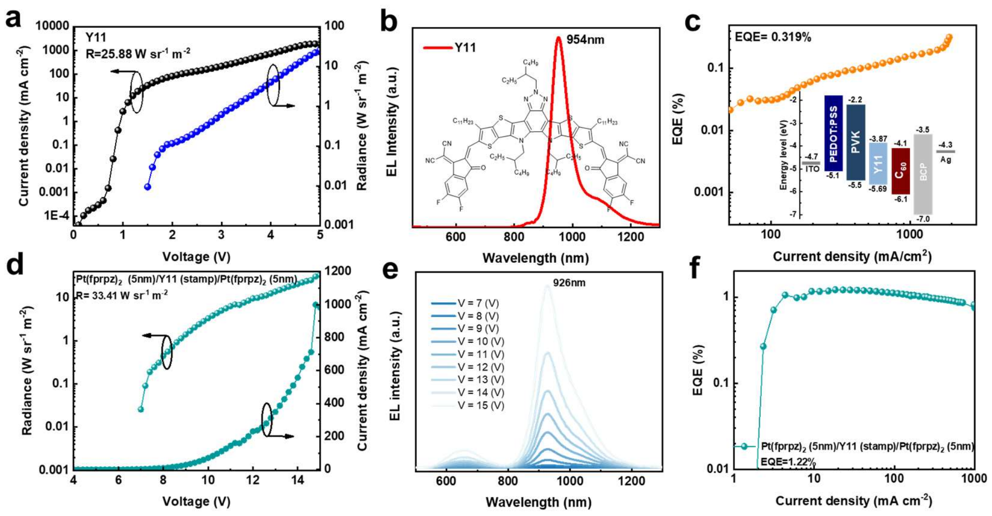

Supplementary Fig. 16 | OLEDs performance measurements. Radiance and current versus voltage curves: (a) Y11 and (d) $Pt(fprpz)_2$ (5nm)/Y11 (stamp)/ $Pt(fprpz)_2$ (5nm). EL spectra of (b) Y11 (inset shows Y11's chemical structure) and (e) $Pt(fprpz)_2$ (5nm)/Y11 (stamp)/ $Pt(fprpz)_2$ (5nm). EQE versus current density curves with device energy level diagram: (c) Y11 and (f) $Pt(fprpz)_2$ (5nm)/Y11 (stamp)/ $Pt(fprpz)_2$ (5nm).

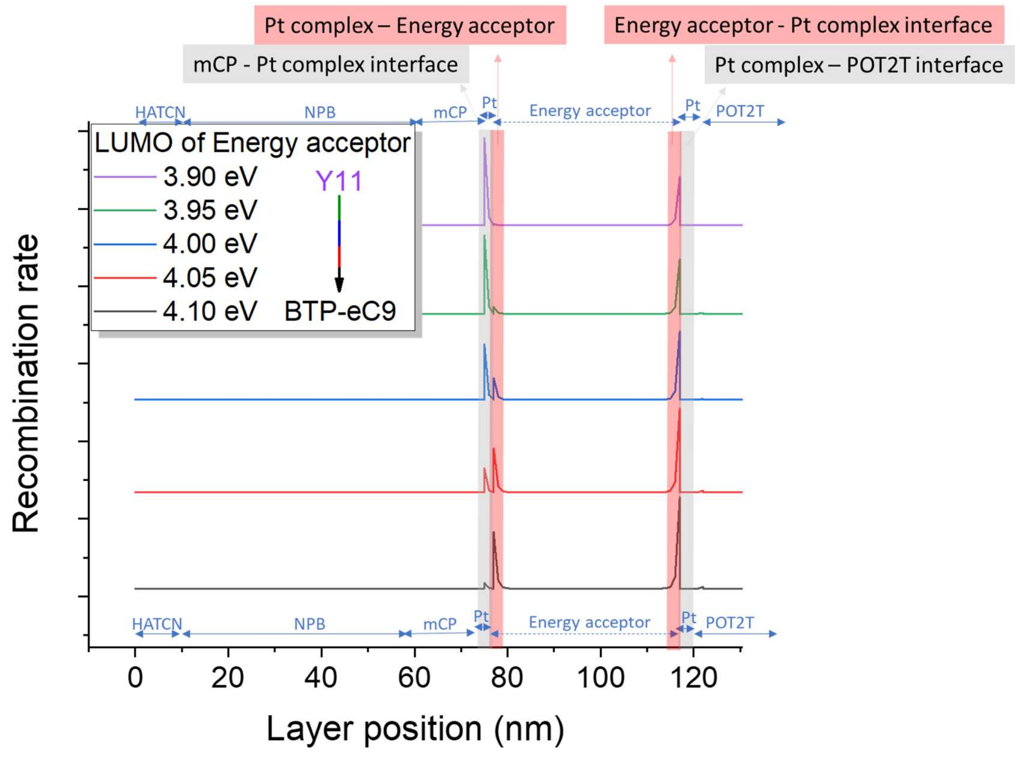

Supplementary Fig. 17 | The simulation results using Setfos. Through simulation calculations using Setfos: Recombination distribution in devices containing altered LUMO of energy acceptor from around -3.9 eV (Y11) to -4.1 eV (BTP-eC9).

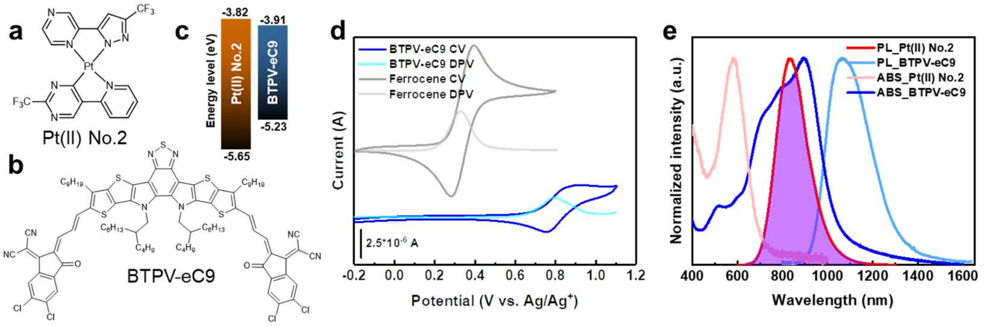

Supplementary Fig. 18 | General information on Pt(II) No.2 and BTPV-eC9. (a) Chemical structure of Pt(II) No.2 and (b) BTPV-eC9 molecules, along with (c) their energy levels. (The HOMO and LUMO of BTPV-eC9 were derived from CV and UV-vis, HOMO and LUMO of Pt(II) No.2 were derived from UPS and UV-vis) (d) CVs and DPVs of BTPV-eC9 in DCM/0.1 M TBAP under nitrogen. (e) Absorption and emission spectra of Pt(II) No.2 and BTPV-eC9, with the overlapping region indicating the radiative energy transfer zone.

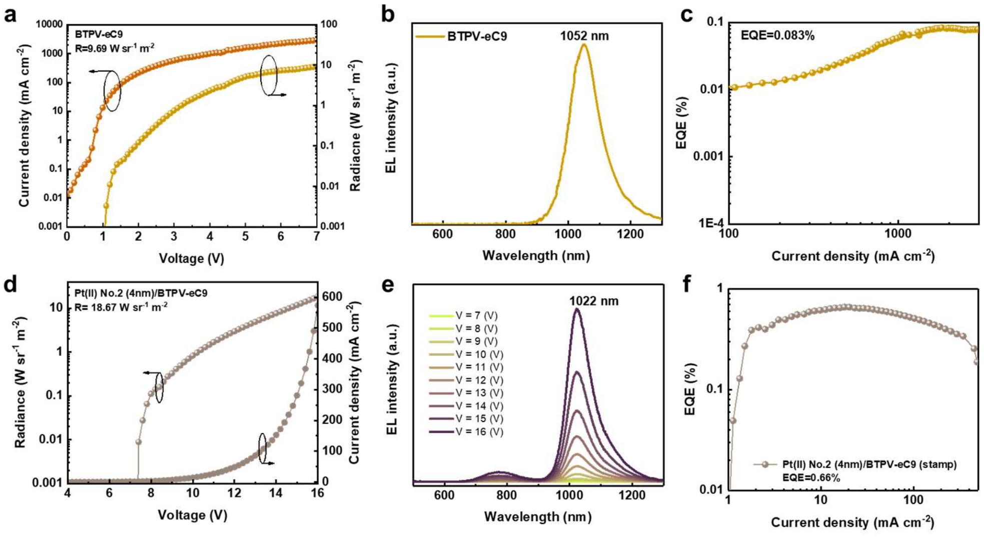

Supplementary Fig. 19 | OLEDs performance measurements. Radiance and current versus voltage curves: (a) BTPV-eC9 and (d) Pt(II) No.2/BTPV-eC9. EL spectra of (b) BTPV-eC9 and (e) Pt(II) No.2/BTPV-eC9. EQE versus current density curves with device energy level diagram: (c) BTPV-eC9 and (f) Pt(II) No.2/BTPV-eC9.

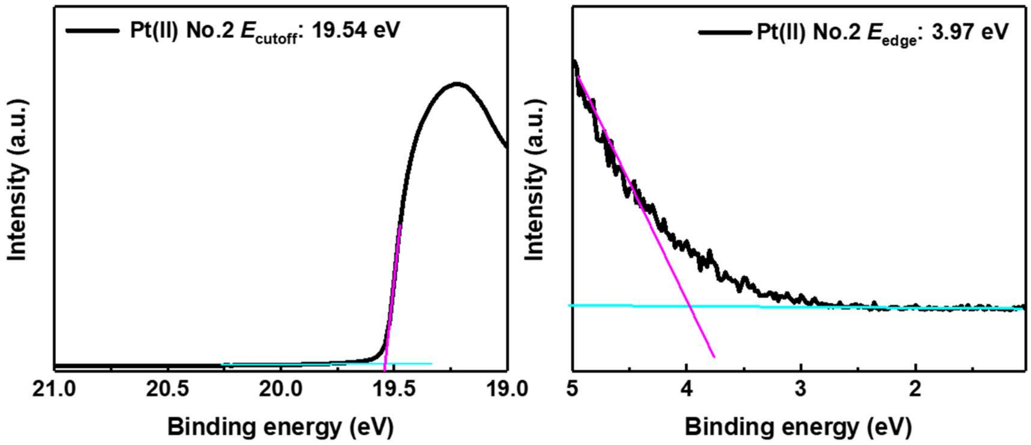

Supplementary Fig. 20 | Determining valence band through ultraviolet photoelectron spectroscopy (UPS). UPS spectra of ITO/Pt(II) No.2 samples.

Supplementary Table 3 | EL performance of Y11, BTP-eC9 and BTPV-eC9 NIR OLEDs.

Emitter	V_{on} (V)	R/J/V (W sr^{-1} m^{-2}/mA cm^{-2}/V)	EQE_{max} (%) (average ± error)	λ_{max} (nm)
Y11[a]	1.5	25.88/1915/5	0.32 (0.23±0.07)	954
Sandwiched (Y11)[c]	7.0	33.41/983/15	1.22 (1.08±0.15)	662 (8.6%), 926 (91.4%)
BTP-eC9[a]	1.3	18.81/2686/4.8	0.18 (0.14±0.04)	952
Sandwiched (BTP-eC9)[c]	6.2	39.97/414/17	2.24 (1.94±0.18)	682 (3.9%), 925 (96.1%)
BTPV-eC9[b]	1.1	9.69/2946/7	0.08 (0.06±0.02)	1052
Bilayer (BTPV-eC9)[d]	7.4	18.67/573/16	0.66 (0.55±0.10)	778 (3.5%), 1022 (96.5%)

The device structure of [a] ITO/PEDOT:PSS/PVK/Emitter/C60/BCP/Ag, [b] ITO/PEDOT:PSS/Emitter/PFN-Br/Ag, [c] ITO/HATCN/NPB/mCP/Pt(fprpz)2/Emitter/Pt(fprpz)2/PO-T2T/LiF/Al and [d] ITO/HATCN/NPB/mCP/Pt(II) No. 2/Emitter /PO-T2T/LiF/Al.

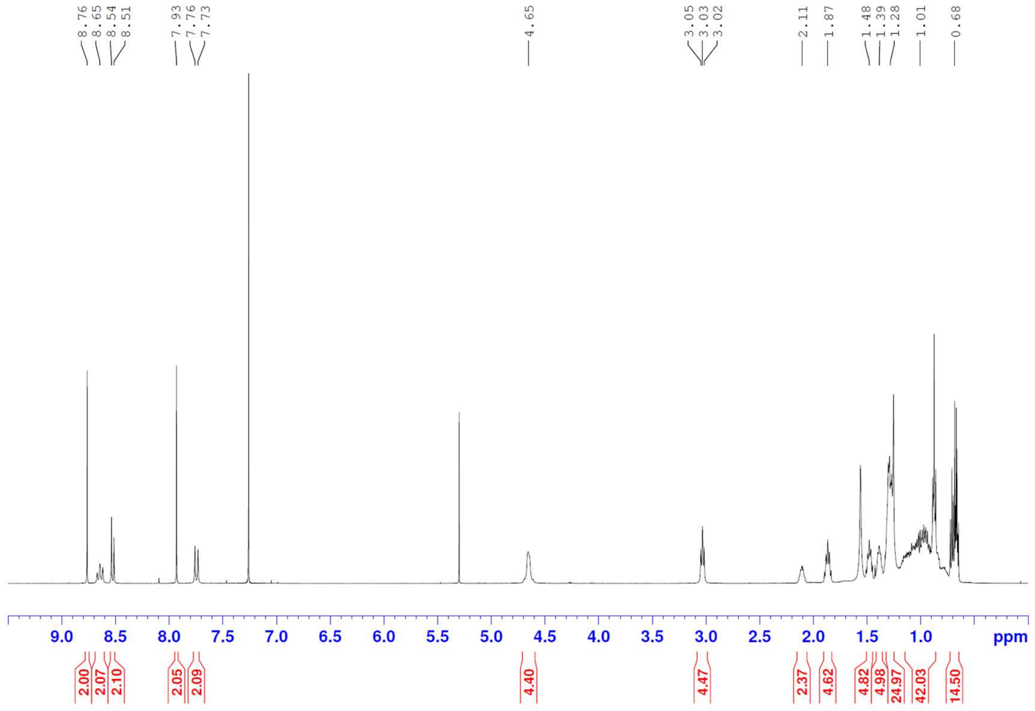

Supplementary Fig. 21 | NMR spectroscopy. ^{1}H-NMR of BTPV-eC9 in $CDCl_3$.

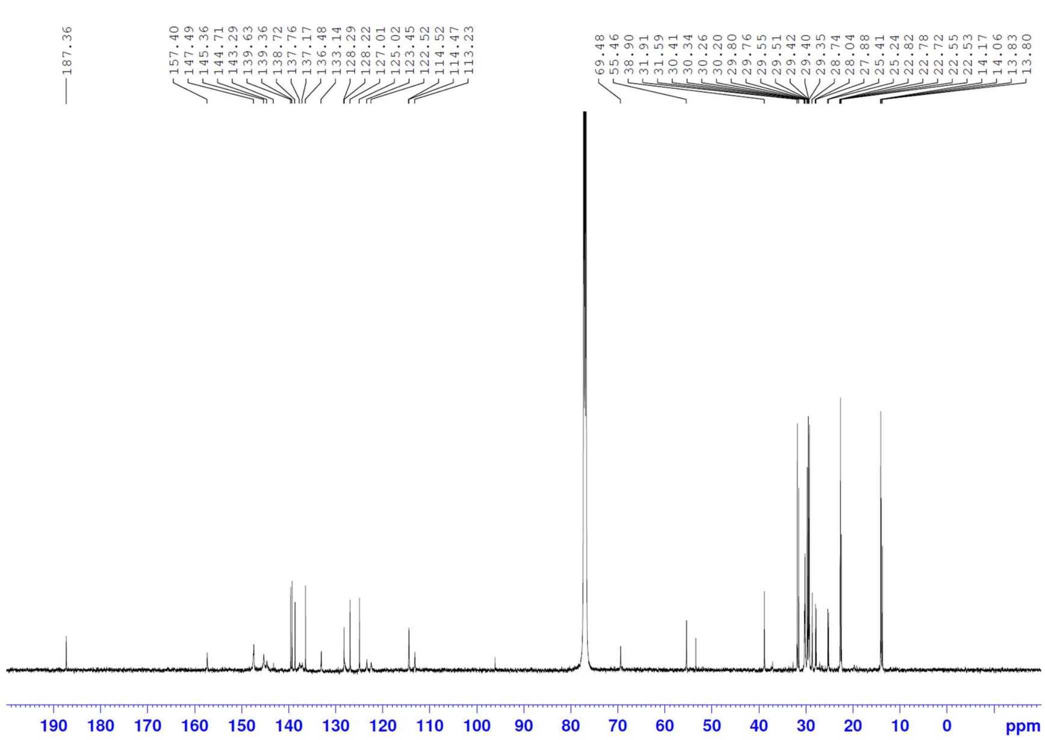

Supplementary Fig. 22 | NMR spectroscopy. ^{13}C-NMR of BTPV-eC9 in $CDCl_3$.

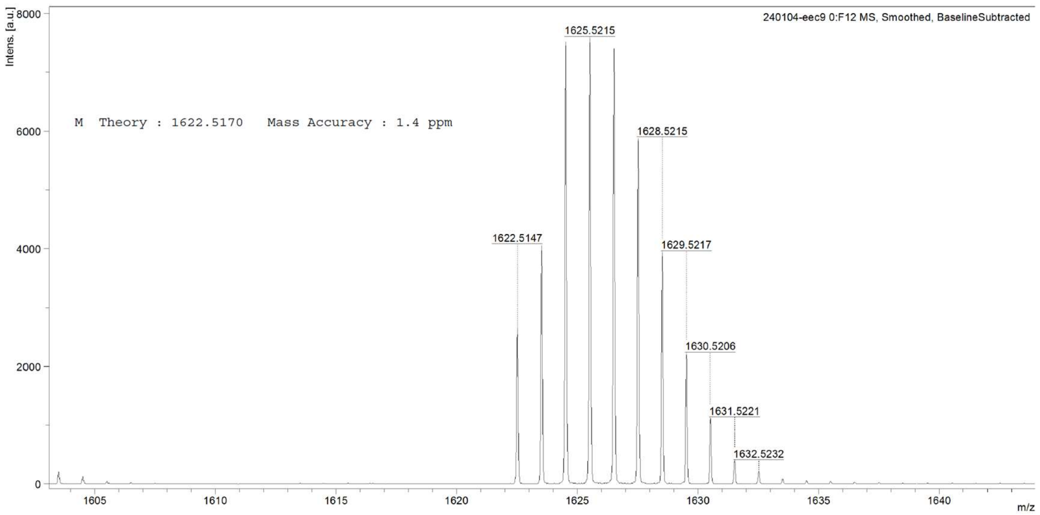

Supplementary Fig. 23 | Mass spectroscopy. High-resolution matrix-assisted laser ionization (HR-MALDI) spectra of BTPV-eC9.

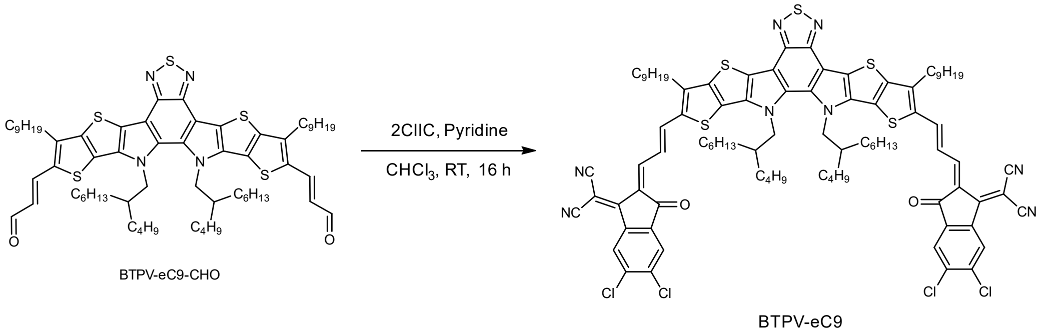

Supplementary Fig. 24 | Synthesis Schematic Diagram. Synthesis route of BTPV-eC9.

Supplementary Reference

1. Qin, S. et al. Non-Halogenated-Solvent Processed and Additive-Free Tandem Organic Solar Cell with Efficiency Reaching 16.67%. *Adv. Funct. Mater.* **31**, 2102361 (2021).
