# Supplementary material for: High-performance near-infrared OLEDs maximized at 925 nm and 1022 nm through interfacial energy transfer
Source: Nat Commun. 2024 May 31;15:4664. doi: 10.1038/s41467-024-49127-x (PMC11143248; doi:10.1038/s41467-024-49127-x)
Supplement: Supplementary file 3 — Description of Additional Supplementary Files [file 41467_2024_49127_MOESM3_ESM.pdf]

File name: Supplementary Movie 1

Description: Demonstration of the spin and stamp techniques and the thin film surface.
